# From distraction to addiction? Understanding academic cyberslacking as a behavioral dependency among medical students

**DOI:** 10.3389/fpsyg.2025.1592370

**Published:** 2026-01-07

**Authors:** Jinyu Zhou, Lifu Jin, Yamin Hu

**Affiliations:** 1 School of Management, Jiangsu University, Zhenjiang, Jiangsu, China; 2 Student Affairs Section, School of Medical Technology, Xi’an Medical University, Xi’an, Shaanxi, China

**Keywords:** academic cyberslacking, medical students, digital distractions, grounded theory, digital behavior

## Abstract

**Background:**

Academic cyberslacking among medical students represents a complex behavioral adaptation shaped by personal, technological, and institutional factors. This study explores its triggers, sustaining mechanisms, and long-term impacts, addressing how digital distractions evolve into entrenched patterns during medical education.

**Methods:**

Using Straussian grounded theory, in-depth interviews were conducted with 35 medical students from two medical universities in China. The analysis followed a structured approach to identify the phased progression of academic cyberslacking behaviors.

**Results:**

Findings reveal a three-phase model. In the trigger stage, students initially engage in digital distractions due to autonomy deficiency, psychological coping, and peer influences. The sustain stage is driven by algorithmic reinforcement, device dependency, and cognitive overload, making disengagement increasingly difficult. Finally, in the impact stage, prolonged cyberslacking leads to motivation decline, cognitive fragmentation, and adaptive digital integration, where students either struggle with disengagement or develop strategic coping mechanisms.

**Conclusion:**

Academic cyberslacking is not merely a distraction but a multifaceted adaptation process. Recognizing its structured progression informs medical education strategies, policy reforms, and wellbeing interventions. The study highlights the need for institutional responses integrating digital literacy, cognitive load management, and technology-aware curriculum design to mitigate public health risks in digitalized learning environments.

## 1 Introduction

In the 21st century, information and communication technologies (ICT) have become deeply embedded in several aspects of human life. The widespread application of this technology is not only a pillar of modern life but also has attracted widespread social attention. Information and communication technologies are like a double-edged sword (Assar et al., 2010; Nobles and Paganucci, 2015) that, while bringing convenience, may also cause a range of problems that affect multiple dimensions of human life (Romeo, 2008). Although universities have benefited extensively from technological advances, tensions have emerged between technology investments and the way students actually use technology. In this study, we define academic cyberslacking as students’ engagement in non-curricular digital activities during scheduled academic tasks or within educational environments. This construct was originally proposed by Keser et al. (2016) and has been further elaborated in the literature as non-academic internet use during lectures or educational activities (Simanjuntak et al., 2022). With the spread of the Internet, students began to use the network provided by universities for personal activities such as gaming, social networking, and shopping, which constitute academic cyberslacking (Stoddart, 2016). The reason for this is that the ubiquity of the Internet enables students to easily access a variety of non-academic content for aimless time-wasting (Lim and Teo, 2005). Students with high confidence in their own media multi-tasking ability often tend to engage in online pastimes during academic activities, such as viewing social media during listening (Wu, 2017; Uzun and Kilis, 2019). Current research shows that, despite increasing investment in technology education, students’ non-academic technology use in educational settings, such as academic cyberslacking, have been relatively neglected. This phenomenon not only affects students’ academic performance but may also affect their mental health and social adaptability (Nobles and Paganucci, 2015; Chu et al., 2017; Reed et al., 2010). Relying on self-supervision is not enough to curb the academic cyberslacking among students, because the digital environment often brings an irresistible temptation (Zhang, 2015; Flanigan and Kiewra, 2018). Studies have shown that inappropriate technology use can lead to student dissatisfaction, distraction, reduced concentration, and psychological and managerial problems (Cidral et al., 2018). With the rapid development of online education, the traditional education model is facing major changes. The rapid rise of various online education platforms has become a new impetus for the education system to maintain stable operations and promote student employment (Shams et al., 2022; King and Boyatt, 2015). These platforms include not only knowledge-intensive online courses and online tutoring but also entertainment-intensive social media and online games, providing students with a comprehensive and diverse range of learning options. However, while online education platforms offer more learning opportunities, they also pose new problems. For example, the lack of a virtual learning environment allows students to engage freely in activities that are unproductive or unrelated to the classroom, so-called “academic cyberslacking” behavior (Varol and Yıldırım, 2017).

In today’s Internet age, college students seeking information, entertainment, and social interaction in cyberspace have become the norm, especially college students, a generation born after 1995 known as “digital natives” who have been exposed to the Internet and digital media since childhood (Seemiller and Grace, 2016). As digital natives, students grow up with the popularity of the Internet, which makes them regard multitasking as a normal aspect of the learning experience (Wu, 2017; Gökçearslan et al., 2016). Nowadays, medical students differ significantly from previous millennials in terms of motivation, learning styles, and social concerns (Rickes, 2016; Ahmady et al., 2020). However, in the digital age, young people regard social media as an important tool for establishing identity and autonomy in social circles. The position they gain from social media enhances their self-concepts (Kumar et al., 2024). Digital environments, including social media, have a stronger influence on students’ attention than traditional classroom environments, especially when teaching methods are considered to lack attractiveness (Gupta and Irwin, 2016; Junco, 2015; Judd, 2013). They have unique characteristics in terms of technology use, social responsibility, and acceptance of diversity (Williams, 2019). Because this generation of students relies heavily on digital technology, their face-to-face communication skills are relatively weak. This deficiency may impair their ability to interact offline with patients and establish personal connections as future healthcare professionals (Baer, 2016; Cretu et al., 2020; Turner, 2015). The monotony of the curriculum and teaching mode may lead to a decrease in students’ participation and attention. Research has shown that when students lose interest or feel bored with course content, they are more likely to turn to academic cyberslacking for immediate stimulation and relaxation (Oliveira and Lathrop, 2022). Furthermore, a curriculum lacking diversity and interactivity makes it difficult for students to maintain long-term motivation and focus (Deslauriers et al., 2019; Exeter et al., 2010). If the students think that the lecturer cannot attract attention or correlation, they are more likely to academic cyberslacking (Varol and Yıldırım, 2017; Alt, 2017). This leads to an increase in academic cyberslacking (Toker and Baturay, 2021; Frick et al., 2020; Dai, 2019). In these cases, students are more likely to seek immediate gratification and relaxation through academic cyberslacking to escape tedious learning tasks (Gasiewski et al., 2012). In today’s highly networked environment, college students often seek information, entertainment, and social interaction in cyberspace, but this also leads to problems such as academic cyberslacking, wasted time, and wasted resources. Academic cyberslacking is considered to be one of the manifestations of ineffective technology use, which may not only reduce students’ attention but also cause problems such as Internet addiction and distraction, which have become the focus of educators’ attention (Akbulut et al., 2016; Yılmaz et al., 2015).

Academic Cyberslacking refers to the behavior of students using the Internet and information technology tools for personal use in an educational environment (O’Neill et al., 2014). Academic cyberslacking includes students’ use of smartphones and other digital devices for non-academic activities during academic time. During academic breaks or unstructured periods, young people often engage in social networking, reading news, gaming, browsing blogs, and shopping (Arabaci, 2017). Peer recognition is a key factor in maintaining academic cyberslacking. This behavior includes accessing news and discussion sites, social network platforms, and other virtual communities, checking email, downloading files (including music), playing online games and shopping online (Yılmaz and Yurdugül, 2018). Academic cyberslacking is common in both work and educational settings and is often considered a waste of time (Weatherbee, 2010). Multitasking has adverse effects on learning, such as cell phone use and texting in class (Rosen et al., 2011), laptop use (Sana et al., 2013), and online messaging (Wang et al., 2012), and may also have a negative impact on performance (Junco and Cotten, 2012; Ravizza et al., 2014). Academic cyberslacking not only affects students’ academic performance but also their mental health and social adaptability (Nobles and Paganucci, 2015; Chu et al., 2017; Reed et al., 2010). On one hand, insufficient attention to students’ academic cyberslacking may lead to increased internet addiction and learning stress (Wu et al., 2018); On the other hand, students may lack long-term learning planning, resulting in a lack of learning motivation and academic achievement (Reed and Reay, 2015; D’Souza et al., 2018). Studies have shown that inappropriate technology use can lead to student dissatisfaction (Attia et al., 2017), distraction (Masood et al., 2020), reduced concentration, and psychological and managerial problems (Hernan et al., 2018). In the current age of Internet popularization, it is normal for college students to seek information, entertainment and social interaction in cyberspace, but this also brings problems such as academic cyberslacking, wasted time and resources. Academic cyberslacking is considered to be one of the manifestations of ineffective technology use, which may not only reduce students’ attention but also may contribute to internet addiction and distraction, which has become the focus of educators’ attention (Akbulut et al., 2016; Barkell et al., 2016). In addition, research has found that academic cyberslacking may be associated with students’ mental health problems, such as anxiety and depression (Maqableh and Alia, 2021). Therefore, it is of great practical significance to deeply understand the factors influencing academic cyberslacking among medical students. Previous studies on students’ academic cyberslacking mainly focus on teenagers’ internet usage habits and mental health, while there are relatively few in-depth studies on the academic cyberslacking behavior of medical students. Although there are a few studies and reviews on medical students in the existing literature, most of them focus on their overall characteristics, and there is still a lack of targeted discussions on the learning styles, preferences, and needs of medical students (Baer, 2016; Dimock, 2019; Howe and Strauss, 2000; Twenge, 2017). Given that current research on the learning needs, perceptions, and learning experiences of medical students remains insufficient, there is an urgent need to further integrate relevant evidence. While they seek convenience and are open to honest feedback, this may also negatively impact the ability to develop offline communication skills and face-to-face interactions with patients in medical practice.

In medical education, where clinical skills and patient interaction are critical, academic cyberslacking not only distracts students from rigorous theoretical learning but also reduces students’ opportunities for hands-on practice and affects patient safety simulations (Pira et al., 2025). These concerns highlight the need to investigate academic cyberslacking among medical students. In addition, medical students need to maintain high professional standards and develop empathy. Constant digital disruption may conflict with ethics training and may reduce student commitment to patient-centered care (Masters et al., 2024). From an institutional perspective, understanding and mitigating academic cyberslacking in medical schools can inform policy and ensure that technology is effectively used for learning rather than undermining clinical capacity (Simanjuntak et al., 2022; Guze, 2015; Aciksoz et al., 2024). This is especially important as educational programs continue to take hybrid or fully online forms. The purpose of this study is to explore the influencing factors of academic cyberslacking behavior of medical students in the educational environment and construct a theoretical model.

## 2 Method

### 2.1 Study design

Based on the content of the study, grounded theory was adopted as the primary qualitative approach for this study (Schwandt, 2007). Developed by Strauss and Corbin, Straussian grounded theory provides a systematic coding and data analysis framework combined with a literature review for a comprehensive understanding of complex social phenomena from different perspectives (Corbin and Strauss, 2014). Unlike Glaser’s classic grounded theory, which focuses on the emergence of new theories, Straussian grounded theory incorporates established theoretical concepts into the analysis (Thornberg, 2012). This methodology is particularly suitable for our study for two main reasons. This methodology is particularly suitable for our study as it facilitates the exploration of the complex academic cyberslacking behavior in medical education, where students, instructors, digital tools, and learning environments interact dynamically. Given the importance of theoretical sensitivity in Straussian grounded theory, we balanced this requirement with inductive grounding by documenting prior assumptions in reflexive memos (Birks et al., 2019; Charmaz, 2014). During open coding we bracketed a formal literature review and privileged *in vivo* codes; theoretical constructs were introduced progressively during axial and selective coding once categories had stabilized, treating prior theory as a comparative resource rather than a template (Corbin and Strauss, 2014). Constant comparison with the raw data ensured that sensitizing concepts sharpened the analysis but did not supplant the inductive model. To capture the students’ vivid experiences of cyberslacking, we conducted semi-structured interviews. COREQ (Comprehensive Criteria for Qualitative Study Reporting) guidelines were followed throughout the study (Tong et al., 2007).

### 2.2 Study context

We conducted this study in the context of Chinese medical universities, where digital technology is widely integrated into students’ academic and daily activities. Medical students have unrestricted access to digital devices and web platforms that are both tools for learning and sources of distraction. Academic cyberslacking occurs in a variety of educational settings, including classrooms, dormitories, and libraries, where students engage in nonacademic online activities. Although universities enforce regulations on digital usage, students retain significant autonomy over their online behavior, resulting in varying degrees of academic cyberslacking.

### 2.3 Data collection and participants

We recruited 35 medical students who had engaged in academic cyberslacking in educational settings such as classrooms, dormitories, or libraries from two separate universities in the northwest of China. We used purposeful sampling to select 30 participants for coding analysis and model building, while the remaining 5 participants were reserved for the theoretical saturation test. The original plan was to randomly select those students who reported academic cyberslacking. However, pilot interviews revealed that the frequency and pattern of academic cyberslacking varied with academic workload and learning environment. Therefore, we prioritized diverse learning habits and digital engagement levels among attendees to ensure that the dataset captured a broad spectrum of academic cyberslacking among medical students.

Data collection involved conducting semi-structured interviews to explore the experiences, motivations, and behavioral patterns of academic cyberslacking among medical students. Participants were asked about “factors affecting their participation in non-academic online activities,” “perceived impact of online relaxation on their academic performance” and “strategies they use to manage digital distractions in their learning environment.” These factors were analyzed based on participant narratives and contextual influences.

Interviews were conducted over a two-month period, specifically in March 2025, with each session lasting 20–30 min. Subsequently, we continued to select and recruit theoretical samples of participants based on theoretical concepts generated from the collected data (Corbin and Strauss, 2014). This iterative process of sampling and analysis is repeated until theoretical saturation has been reached, which means that no new significant categories have emerged and no additional data collection is required (Babchuk, 2016).

Data collection and constant comparative analysis proceeded concurrently. We predefined theoretical saturation as the point at which no new open codes emerged, the properties or dimensions of existing categories stabilized, and no new relationships were identified during axial or selective coding, consistent with grounded theory guidance (Glaser and Strauss, 1967; Saunders et al., 2018). After analyzing 30 interviews, coding memos indicated redundancy across major categories; we then conducted 5 additional interviews as a saturation probe. These confirmatory cases yielded no novel codes, properties, or inter-category linkages, and we judged theoretical saturation achieved. To enhance credibility, we conducted member checking by returning thematic summaries with exemplar quotations to a purposive subset of participants (*n* = 8); all confirmed interpretive fit, and minor clarifications were incorporated (Glaser and Strauss, 1967). For dependability and confirmability, two independent qualitative methodologists (one >10 years’ grounded theory experience; one medical education researcher trained in qualitative analysis) audited coding outputs, category definitions, and the saturation decision; discrepancies were resolved by consensus, and an audit trail of analytic memos was maintained (Keshmiri, 2025).

### 2.4 Data analysis and trustworthiness

The collected data were analyzed in terms of the influencing factors of academic cyberslacking behavior. The recorded interview file and the researcher’s notes were transcribed using iFlyrec 3.0 (iFlyrec Tingjian), with manual annotation, correction of spelling errors, and semantic adjustments. Non-verbal cues such as pauses, silence, laughter, and intonation were inserted into the transcribed text. The data were then analyzed by grounded theory using the qualitative research software NVivo 12 (Windows), where all collected data were systematically categorized into concepts through line-by-line analysis to represent underlying ideas and phenomena.

To ensure validity and reliability, multiple validation strategies were applied throughout the data analysis process (Babchuk, 2016). Systematic comparison with existing literature, the study aims to align new findings with established theoretical frameworks in order to identify key attributes and dimensions that may have been overlooked (Corbin and Strauss, 2014). To enhance methodological rigor, expert reviews were conducted by two experts, one with extensive experience in qualitative research and fundamental theoretical methodology, and the other with expertise in digital behavior and medical education. These experts independently evaluated coding procedures, category structures, and theoretical explanations to confirm that the results were methodologically sound and accurately represented participants’ experiences. Member check (participant validation) ensures that their experience is faithfully reflected by providing participants with a summary of preliminary findings and asking them to provide feedback on the accuracy of the interpretation. This iterative feedback process helps refine the category definition and increase the credibility of the study. In addition, five additional interviews were conducted on a theoretical saturation test. No new core classes or class relationships emerged in the stratified analysis, confirming that the model had reached theoretical saturation and no further data collection was necessary.

### 2.5 Ethics statement

This study was approved by the Medical Ethics Review Committee of Xi’an Medical University (Approval No. XYLS2025006). Prior to the interview, participants received a thorough explanation of the purpose of the study and the content of the interview. They were then asked to sign a “Research Participation Consent Form,” and only after their consent was obtained would the interview proceed.

## 3 Results

We interviewed 35 medical students who were undergoing their medical training at two different universities. Table 1 provides their detailed demographic characteristics. According to participants’ statements, their academic cyberslacking was influenced by multiple interrelated factors that shaped their exposure to digital distractions in educational and clinical settings. As they receive their medical education, they experience shifts in motivation, social interaction, and technology use, culminating in habitual patterns of academic cyberslacking.

**Table 1:** Participants’ characteristics.

Variables	Category	*n*
Sex	Female	17
	Male	18
Mean age (Years)	20.4	35
Age range	18–23 years	35
Urban/Rural origin	Urban	16
	Rural areas	19
Daily screen time	<4 h	4
	4–10 h	21
	>10 h	10
Medical school	Western medicine-oriented medical schools	15
	Traditional Chinese medicine (TCM) medical schools	20
Year	Medical 1	7
	Medical 2	8
	Medical 3	6
	Medical 4	6
	Intern	6
Specialty	Radiology	5
	Obstertics-gynecology	4
	Surgical specialty	3
	Pathology	4
	Herbal medicine	5
	Acupuncture and moxibustion	3
	TCM orthopedics	4
	TCM internal medicine	3
	TCM surgery	4

Table 2 shows the themes and sub-themes extracted from the analysis, which are discussed below.

**Table 2:** Examples of participants’ comments on the phases of academic cyberslacking among Chinese medical students.

Categories	Sub-Categories	Quotes
Phase 1: trigger stage		
Individual drive	Autonomy deficiency	I set a goal to study for an hour before taking a break, but I always end up scrolling through social media after just 10 min. (Participant A)
	Psychological compensation	Whenever I feel overwhelmed with coursework, I take ‘just a few minutes’ on my phone to clear my mind—but that break always lasts longer than I intend. (Participant C)
	Physiological Compensation	Whenever I pull an all-nighter, my brain feels foggy the next day, and I end up wasting time on my phone rather than being productive. (Participant G)
Social and environmental influences	Social learning effects	I used to feel guilty about checking my phone during lectures, but when I saw my classmates doing it openly, I started doing the same. (Participant D)
	Norm deconstruction	Some professors act like they do not notice us using our phones in class. Over time, it felt like an unspoken rule that it was okay. (Participant B)
	Teaching interaction gap	The professor reads from slides the whole lecture. I do not see the point in paying attention when I can just download the notes and review later. (Participant F)
	Attention competition	Every few minutes, my phone lights up with notifications from different apps. I try to ignore them, but eventually, I give in and check.” (Participant G)
Phase 2: sustain stage		
Technological factors	Algorithmic reinforcement	I only wanted to watch one medical case video, but the algorithm kept recommending more interesting, unrelated content. I lost track of time. (Participant E)
	Device dependence	If I do not have my phone next to me, I feel uneasy, like I’m missing something important. (Participant S)
Digital entrapment	Self-control depletion	After a long day in the hospital, I have no energy left to fight distractions. I just let myself scroll endlessly.” (Participant H)
	Cognitive load saturation	Too much medical information at once makes me feel burned out, and I turn to my phone to escape.” (Participant K)
	Digital loop reinforcement	I check my phone for a short break, but the content keeps me hooked, and I keep coming back. (Participant I)
Phase 3: impact stage		
Goal erosion	Motivation decline	At first, I was excited about learning medicine, but now I feel like I’m just surviving each day. Watching videos seems more appealing than studying.” (Participant M)
	Reward uncertainty	Studying feels like an endless process with no immediate reward. At least social media gives me instant gratification. (Participant P)
Cognitive overload	Information fatigue	Every day, there’s a new guideline or protocol to learn. My brain feels exhausted, and I turn to cyberslacking to reset. (Participant N)
	Decision paralysis	Choosing which resources to use is overwhelming, so I procrastinate by browsing online instead. (Participant R)
	Cognitive fragmentation	Switching between studying and checking my phone makes it hard to concentrate. I feel like my brain is constantly being interrupted. (Participant S)
Adaptive digital integration	Selective cyberslacking	I only slack off by reading medically related articles, so I do not feel guilty. But in the end, I still waste time. (Participant F)
	Compensatory learning strategies	I tell myself that as long as I study efficiently later, taking frequent breaks for cyberslacking is fine. (Participant T)
	Boundary regulation	I’ve set strict rules for myself: no social media before finishing my daily study goals. (Participant M)

### 3.1 The evolution of academic cyberslacking among medical students

The findings of this study pertained to the factors influencing academic cyberslacking among medical students. These influencing factors were categorized into four overarching themes: “Individual Drive,” “Social & Environmental Influences,” “Technological Factors” and “Goal Erosion.” Individual drive involved aspects related to self-regulation, physiological compensation, and motivational changes. Social and environmental influenced included peer norms, institutional culture, and educational expectations. Technological factors featured the role of algorithmic reinforcement, device dependency, and digital distractions. Goal erosion addressed the weakening of academic motivation and uncertainty regarding long-term rewards. Participants described experiences that shaped their engagement in academic cyberslacking, highlighting how these factors interact to sustain or intensify academic cyberslacking over time. The specific manifestations of these influencing factors, as expressed by participants, are discussed in the following sections. Table 2 summarizes the key themes along with representative participant quotes.

#### 3.1.1 Triggers of academic cyberslacking

##### 3.1.1.1 Individual drive

###### 3.1.1.1.1 Autonomy deficiency

The lack of autonomy meant that as participants adjusted to the strict academic demands of medical education, their ability to accommodate digital distractions gradually declined, leading to increased slack in academic cyberslacking. At first, they tried to maintain a strict academic routine, but as academic pressure increased, they found themselves increasingly drawn to nonacademic online activities. One participant described setting a timer to focus on learning, but often unconsciously checking his phone to disrupt their concentration during lessons (Participant A). Another interviewee noted that they usually start by browsing lecture slides but end up browsing unrelated websites, unable to resist the urge to multitask. Over time, these lapses become habituated, making it increasingly difficult to maintain focus on academic tasks.

###### 3.1.1.1.2 Psychological compensation

Psychological compensation refers to participants’ tendency to engage in academic cyberslacking as an emotional escape from academic stress. While this provides temporary relief, it ultimately interferes with their ability to concentrate on learning. Many participants reported seeking comfort on social media or entertainment platforms after stressful exams or long hours of study, even though they knew it was an unhelpful habit. One participant shared that after a stressful day of clinical training, they instinctively turned to short-video platforms to relax, even though they knew too much screen time would make them feel more tired than refreshed (Participant C). Over time, participants increasingly relied on digital distractions to avoid facing stress, which impaired their ability to develop healthier coping strategies.

###### 3.1.1.1.3 Physiological compensation

Physiological compensation describes how participants turned to online activities to offset physical fatigue caused by their demanding schedules. This behavior was initially thought of as a way to relax, but ultimately interfered with learning efficiency. Because participants endure long periods of study and clinical rotation, they often resort to digital engagement to temporarily relieve fatigue. One participant admitted that after studying late at night, they routinely browsed social media or streamed videos before sleep, believing it to alleviate stress but this often led to a decline in sleep quality (Participant E). Over time, this behavior formed a vicious cycle, where digital distractions provided short-term fatigue relief but disrupted their sleep patterns, further reducing their academic performance and overall well-being.

##### 3.1.1.2 Social and environmental influences

###### 3.1.1.2.1 Social learning effects

Participants described how social learning processes led them to normalize academic cyberslacking through peer observation and imitation. At first, many students stuck to self-imposed academic discipline, but their perceptions of acceptable behavior changed when they saw classmates casually browsing social media or engaging in online entertainment. Over time, normalization of academic cyberslacking among peer groups causes participants to adopt similar behaviors, often without awareness. One participant recalled feeling guilty about checking their phones in class when they first entered medical school. However, after noticing that many of their classmates did the same, they stopped thinking it was inappropriate and even started using online forums to follow unrelated discussions during lectures (Participant G). This peer-reinforcement mechanism makes academic cyberslacking a common behavior rather than an individual behavior, reinforcing its persistence among student groups.

###### 3.1.1.2.2 Norm deconstruction

Normative deconstruction refers to the gradual breakdown of previously established academic expectations, allowing cyberslacking to become increasingly embedded in students’ daily learning environments. It was noted that traditional learning environments that once required intense concentration had been transformed into digital spaces in which multitasking had become the accepted norm. As online participation becomes a regular part of their learning experience, the line between academic activity and leisure becomes blurred. One participant described the break time for the lecture, which was once used to review notes and gradually became a designated time to scroll through social media messages or view e-commerce sites (Participant M). The erosion of strict academic norms means that behaviors that were once seen as distractions are now seen as acceptable learning habits, making it harder for students to recognize and resist academic cyberslacking.

###### 3.1.1.2.3 Teaching interaction gap

The limited instructional engagement exacerbates academic cyberslacking by reducing student engagement and motivation during lectures. Participants often cited passive lecture formats, outdated teaching methods, or limited professor-student interaction that left them feeling disconnected from the learning process. In the absence of dynamic discussion or interactive learning, students often turn to digital platforms as alternative sources of stimulation. One participant described how they initially tried to take notes, but over time unconsciously turned to messaging apps or news sites when the lecture lacked participation (Participant C). The lack of interaction and real-time feedback from teachers leads to online slacking as the default response to passive learning experiences in academic settings.

###### 3.1.1.2.4 Attention competition

The competition between academic materials and external digital stimuli for students’ attention is the main reason for the relaxation of academic networks. In the face of constant notifications from social media, news updates and entertainment platforms, participants described their struggle to maintain attention. The constant digital presence in their learning environment places a significant cognitive load on students, often diverting attention from lectures or learning materials. One participant admitted that despite the intent to focus on coursework, the temptation to check for incoming notifications often interfered with their attention, resulting in prolonged online distraction (Participant K). The interplay between external digital stimuli and the inherent difficulty of sustained academic concentration makes cyberslacking an almost inevitable behavior.

Social learning effects, normative deconstruction, instructional interaction gaps, and attentional competition collectively illustrate how social and environmental influences positively shape the persistence of academic cyberslacking. The combination of peer reinforcement, changing academic norms, passive learning environments, and digital distractions creates a culture in which cyberslacking becomes normalized and even expected.

#### 3.1.2 Sustenance of academic cyberslacking

##### 3.1.2.1 Technological factors

###### 3.1.2.1.1 Algorithmic reinforcement

Algorithmic reinforcement refers to the fact that as participants continue to engage with digital platforms, recommendation algorithms increasingly personalize their content, making it increasingly difficult to disengage from non-academic material. Initially, participants intended to take short breaks by casually browsing social media or watching videos. However, due to algorithmic curation, they are repeatedly exposed to content that is more attractive and aligned with their interests, resulting in long-term digital engagement. Specifically, they report that searching for academic material alone can quickly lead to unrelated entertainment recommendations, significantly extending their screen time. One participant shared that after watching a medical course, their stream was flooded with entertainment videos, which made it harder for them to refocus on learning (Participant F). Over time, this algorithm-driven mode of engagement reinforces habitual reliance on digital platforms, making academic cyberslacking an entrenched behavior rather than an occasional distraction.

###### 3.1.2.1.2 Device dependence

Device dependency means that as participants increasingly rely on smartphones, tablets and laptops for academic work, they are close to these devices for habitual access to non-academic content. Specifically, they reported that learning from nearby devices made it difficult to resist the urge to check notifications, browse social media, or engage in recreational activities. One participant noted that even in focused learning sessions, the mere presence of a phone prompted them to pick up their phone and browse social media without realizing it (Participant D). This habitual device checking blurs the line between academic work and online leisure, creating an environment in which digital distraction becomes an expected and persistent aspect of learning routines. Over time, this continued accessibility leads to cycles of academic disengagement, reinforcing the long-term impact of technological factors on cyberslacking behavior.

##### 3.1.2.2 Digital entrapment

###### 3.1.2.2.1 Self-control depletion

Self-control depletion means that as participants engage more in digital distractions, their ability to regulate online behavior diminishes, exacerbating the cycle of cyberslacking. Initially, participants struggled to resist the lure of social media or entertainment platforms, but as academic workloads increased, their willpower weakened, making avoidance increasingly difficult. One participant reported that although they initially set a strict learning schedule, they found themselves repeatedly succumbing to digital distractions, even though they were fully aware of their negative effects (Participant A). Over time, their self-regulatory mechanisms deteriorate, leading them to habitually engage in nonacademic online activities despite their conscious focus on learning.

###### 3.1.2.2.2 Cognitive load saturation

Cognitive load saturation refers to excessive mental stress that prevents participants from paying sustained attention to academic tasks, thereby increasing reliance on digital distractions as coping mechanisms. Participants said they felt mentally exhausted trying to remember information or finish assignments efficiently after long hours of study. As a result, they unconsciously turn to passive digital content as a means of cognitive relief. One participant noted that after spending hours memorizing medical terms, their brains felt “overloaded to function,” prompting them to browse social media as a mental escape (Participant C). This habitual use of digital engagement to counteract cognitive fatigue further deepens the cycle of network fatigue.

###### 3.1.2.2.3 Digital loop reinforcement

Digital loop reinforcement means that participants described being caught in a feedback loop in which initial engagement with digital distractions triggers algorithmic feedback loops that make it increasingly difficult to disengage. Participants reported that once they interacted with entertainment or social media platforms, recommendation algorithms kept pushing similar content, reinforcing their habitual scrolling behavior. One participant shared that after watching a short video during breaks, they found themselves “unconsciously consuming” the entire suggested video, which made it difficult for them to restart learning (Participant E). The seamless transition from one type of disturbance to another creates a reinforcement mechanism that sustains cyberslacking beyond the participants’ initial intent.

#### 3.1.3 Impact of academic cyberslacking

##### 3.1.3.1 Goal erosion

###### 3.1.3.1.1 Motivation decline

Declining motivation meant that as participants increasingly engaged in online relaxation behaviors, their intrinsic motivation to excel in academic work waned. Initially, participants showed a strong academic desire, but frequent engagement with digital distractions slowly weakened their commitment to learning. Specifically, they reported that prolonged exposure to relaxed digital entertainment reduced their willingness to participate in complex or cognitively demanding classes. One participant shared that after repeatedly using Short Video as a quick break, they found themselves lacking motivation to return to learning, often dragging it to the last minute (Participant G). Over time, the decline in motivation to learn reinforced a pattern in which participants associated learning with mental fatigue and internet access with instant gratification, deepening their disconnect from academic responsibility.

###### 3.1.3.1.2 Reward uncertainty

The uncertainty of rewards means that as participants struggle through a long and rigorous medical education process, the perceived gap between effort and actual reward leads to their persistent online slacking behavior. Unlike the immediate feedback of digital interaction, academic achievement requires long-term investment and delayed gratification, which makes the perceived value of learning less attractive. Specifically, participants reported that the slow pace of academic progress made online relaxation more attractive because it provided immediate stimulation without effort. One participant reported that after weeks of intensive learning without immediate academic success, browsing entertainment content felt more rewarding and fulfilling (Participant K). This growing disillusionment with the delayed rewards of medical education led them to rely increasingly on digital distractions as coping mechanisms, ultimately undermining their long-term academic engagement.

##### 3.1.3.2 Cognitive overload

###### 3.1.3.2.1 Information fatigue

Participants described mental exhaustion due to prolonged exposure to intensive medical content, resulting in increased reliance on digital distractions. Long study sessions are filled with medical textbooks, clinical guidelines and research articles, making it difficult for them to remember key information. One participant noted that after reviewing pharmacology for hours, they instinctively browsed social media and were unable to process any more complex material (Participant G). As digital engagement increases, their ability to absorb academic content diminishes, making it more difficult to maintain an effective learning curriculum.

###### 3.1.3.2.2 Decision paralysis

The number of academic responsibilities and numerical choices often prevented participants from making valid learning choices. Instead of prioritizing important topics, they fall into indecision, often turning to entertainment as an escape. One participant recalled turning on multiple medical resources to prepare for the exam but ended up watching an unrelated Short Video because he could not decide where to start (Participant M). As this pattern repeats, participants find it increasingly difficult to keep track of academic priorities, which exacerbates their reliance on digital distractions.

###### 3.1.3.2.3 Cognitive fragmentation

Frequent digital interruptions interfered with participants’ ability to stay focused on academic tasks. Notifications, messages, and social media hustle distract them, making deep learning difficult. One participant shared that constant phone checks forced them to reread chapters multiple times while reading patient records, delaying their ability to form diagnostic reasoning (Participant T). Over time, these interruptions reinforce attention cycles, further weakening their ability to stay focused for long periods of time.

##### 3.1.3.3 Adaptive digital integration

###### 3.1.3.3.1 Selective cyberslacking

Selective cyberslacking refers to students intentionally engaging in digital distractions to manage cognitive fatigue while maintaining learning efficiency. At first, participants thought academic cyberslacking was harmful, and they often felt guilty when they spent time on nonacademic online activities. However, as medical education progressed, they began to recognize the benefits of controlled digital engagement. Instead of blindly browsing social media, they strategically use short-lived digital distractions, such as short videos or games, to refocus their attention between study sessions. One participant noted that watching a five-minute video after an intense study helped them refocus back on the task (Participant D). Over time, this shift from impulsiveness to structured cyberslacking indicates that students can regulate their digital engagement rather than being passively dominated by it.

###### 3.1.3.3.2 Compensatory learning strategies

In addition to strategic online relaxation, students are using digital learning tools to compensate for missed academic progress. At first, participants expressed anxiety over academic setbacks caused by excessive online distraction. However, they adapt their learning habits by integrating digital platforms such as recorded lectures, AI-powered note summaries, and educational videos. Instead of struggling to keep pace with traditional learning methods, they use these online resources to efficiently reinforce key concepts. One participant reported that after realizing they spent an hour on social media, they quickly turned to medical tutorials on YouTube to make up for lost time and reinforce learning (Participant B). This ability to recover from distractions highlights that digital engagement is not inherently detrimental but may serve as a complementary reinforcement mechanism.

###### 3.1.3.3.3 Boundary regulation

As students became more aware of their digital habits, they implemented strategies to establish clear boundaries between academic tasks and online leisure. At first, many people struggle with self-control and often lose awareness of time while engaging with entertainment content. Over time, they developed self-imposed digital rules, such as timed study tech breaks, and applied limit or focus mode settings to manage screen time without completely eliminating digital engagement. One participant shared that setting an app timer helped them limit their use of social media during study time while still allowing them to relax online during designated breaks (Participant F). These practices reflect an evolving form of digital self-regulation and that students are actively regulating their digital behavior rather than aiming to eliminate cyberslacking entirely.

## 4 Discussion

From a behavioral perspective, this study explored how medical students develop patterns of academic cyberslacking and what sustaining and long-term effects emerge through this behavior. Based on the results, the triggers that initiate cyberslacking, the factors that sustain it, and the impact it generates can be discussed below.

Academic cyberslacking of medical students is a complex and changing phenomenon, which is formed by the interaction of individual, environmental and technical factors. As these behaviors progress, they profoundly affect students’ academic engagement, cognitive ability, and goal orientation in the context of rigorous medical education. Understanding these dynamics is critical to determining their impact on learning efficiency and professional growth. While existing research has primarily examined the prevalence and immediate consequences of online relaxation in a broader educational setting, the unique challenges medical students face remain underexplored. The interaction between intense learning schedules and constant digital distractions requires deeper investigation. This study reveals the mechanism of academic cyberslacking in three interrelated stages: its triggers, persistence mechanisms and long-term effects. By analyzing how these stages interact with students’ academic and cognitive processes, this study reveals the multifaceted nature of online relaxation and its impact on educational strategies. Addressing these behaviors as a whole has the potential to strengthen academic regulation and support professional development in medical education.

First, Trigger-level factors reflect how individual vulnerabilities, social dynamics, and contextual environments converge to initiate cyberslacking behavior. These dynamics reflect how individual vulnerabilities, peer influence, and digital environments converge to trigger online disruptions in academic environments. The interplay of these factors highlights a complex phenomenon that goes beyond simple waste of time and reveals deeper psychological and social motivations behind online relaxation behavior. Lack of autonomy is an important driver of academic cyberslacking, as students struggle to maintain self-discipline amid demanding academic schedules. This pattern aligns with the core proposition of Self-Determination Theory, which holds that when students experience autonomy-need frustration due to externally imposed schedules and limited agency in learning, they are more inclined to seek self-directed digital activities to regain a sense of control (Ryan and Deci, 2020). Studies have shown that individual psychological factors, such as self-regulation and emotional stability, significantly affect online behavior and mental state (Pellas, 2014). One participant noted, “I tried to stick to my study schedule, but ended up checking my phone unknowingly” (Participant A). Psychological compensation has also become a recurring theme, with students using network interference as a coping mechanism to escape the stresses and strains of medical education. This aligns with transactional models of stress and coping, which propose that individuals experiencing uncontrollable academic stress often adopt emotion-focused strategies such as digital browsing to relieve pressure when problem-solving feels ineffective (Lazarus, 1984). This is in line with the principle of immediate gratification theory (Steel and König, 2006), which suggests that individuals tend to prioritize behaviors that provide immediate relief from discomfort over behaviors that require delayed effort and reward. Medical students may learn because of cyberslacking when facing academic stress, although they recognize the long-term disadvantages of cyberslacking. Past research has shown that emotional escape via digital platforms tends to interfere with concentration and exacerbate academic disconnect (Osei et al., 2022). For example, one participant explained: “After exams, I browse social media, even though I know it will not help me relax-it’s just to avoid thinking about studying” (Participant C). This reflects previous findings that lower emotional regulation leads to greater reliance on network activity to relieve stress (Park et al., 2014). In addition, physiological compensation is thought to be another trigger, with students resorting to digital distractions to relieve physical fatigue. This behavior is consistent with studies highlighting how poor sleep quality and physical fatigue lead to habitual late-night Internet use (Kim et al., 2018; Xanidis and Brignell, 2016). One participant shared: “After long clinical hours, I use my phone to relax before bed, but it often makes me lose more sleep” (Participant E). This cyclical behavior exacerbates fatigue and further reduces learning, concentration and performance (Moulin, 2015). Peer influence plays a key role in normalizing cyberslacking, as students observe and mimic peer network interference. According to Bandura’s social learning theory, individuals tend to adopt behaviors they see modeled and rewarded within their social group (Bandura, 2001). In this study, witnessing classmates use phones during lectures appeared to shift normative boundaries, subtly legitimizing academic cyberslacking. This is consistent with *social encouragement theory* (Raymen and Smith, 2020), this study postulates that individuals are more likely to engage in certain behaviors when they believe they are socially accepted or encouraged in peer groups. This is consistent with research showing that social learning effects, coupled with peer behavior, drive exploratory online activity (Gulzar et al., 2022). One participant said, “At first, I did not use my phone when I was in class, but when I saw other people doing it, I started to think it wasn’t a big deal” (Participant G). This suggests that medical students may develop the habit of being lazy online, not only because of personal motivation, but also because they see similar behavior in their peers, which reinforces their normality in academic settings. This shows how social modeling embeds cyberslacking into group dynamics, as previous research has shown that positive reinforcement from Social networks increases online engagement (Gulzar et al., 2022). The deconstruction of traditional academic norms further blurs the line between academic and non-academic activity. One participant observed that “during breaks, everyone did not review notes, but browsed their phones” (Participant M). This shift in norms has allowed internet slackness to be seen as an acceptable habit rather than a form of disruption (Paul et al., 2012). The teaching interaction gap was also prominent, with students citing passive lecture styles and limited teacher engagement as contributing factors. Research has shown that positive instructional interactions significantly improve students’ attention span and reduce unnecessary Internet use (Liu and Wu, 2023). One participant shared, “When the lecture was monotonous, I found myself browsing social media just to stay engaged” (Participant C). These findings highlight the need for dynamic and interactive teaching methods to reduce disengagement and mitigate cyberslacking. Finally, competition for attention is a key driver, as digital notifications and external stimuli constantly compete for students’ cognitive resources. This finding is consistent with previous research showing that external distractions reduce students’ concentration on academic tasks (Van Nuland et al., 2017). One participant admitted that “even with good intentions, notifications pull me back to my phone” (Participant K). This aligns with *technology-mediated behavioral addictions* (Griffiths et al., 2017), which suggests that persistent exposure to digital content, especially algorithm-driven recommendations, reinforces compulsive technology use. These distracting dynamics highlight the pervasive effect of numerical stimuli on learning environments, further reinforcing online relaxation behavior.

Second, Sustain-stage factors center on the technological affordances and feedback mechanisms that maintain and reinforce cyberslacking over time. Algorithm reinforcement, device dependency, and digital traps create an environment that makes it difficult for students to disengage from nonacademic content, thereby shaping their learning behavior in complex ways. The algorithmic reinforcement mechanisms observed in cyberslacking and behavioral addiction mediated by technology suggest that continuous exposure to algorithmically driven digital environments increases compulsive engagement. Medical students report that algorithm-driven recommendations intensify their exposure to nonacademic content, making self-regulation more difficult. Initially, students sought short online breaks, but personalized content streams quickly expanded their engagement. Many interviewees noted that recommendations quickly shift from a short medical clip to an endless string of highly appealing entertainment, extending a planned two-minute break into a 20-min scroll. One participant described how, after watching a medical course, their video stream was flooded with unrelated entertainment content, which made it harder for them to return to learning (Participant F). Such snowballing engagement is typical of persuasive-technology loops, where high-frequency cues and variable rewards cement automatic habits (Fogg and Fogg, 2009; Wood and Neal, 2007). This is consistent with research showing that algorithmic systems optimize engagement by presenting increasingly stimulating content, reinforcing habitual online behavior (Van Nuland et al., 2017; Song et al., 2018). Unlike previous research that only focused on excessive screen time, recent research has shown that the structure of digital platforms, especially their predictive recommendation systems, plays a crucial role in digital traps (Hassenzahl and Tractinsky, 2006). Solving this problem requires developing algorithmic interventions that prioritize academic content while limiting exposure to interference. Adaptive digital environments, such as AI-based learning models that adjust recommendations based on academic relevance, may help mitigate the negative effects of algorithmic hardening. Academic reliance on personal devices blurs the line between learning and leisure, making it harder for students to resist digital temptations. Attendees often noted that their proximity to smartphones and laptops led them to habitually check notifications and social media, even during focused learning sessions. One participant shared that the mere presence of the phone triggered auto-scrolling behavior that kept them away from schoolwork (Participant D). Attention research shows that ever-present cues such as screen light, vibrations, and notification tones trigger approach impulses, especially when self-control resources are low (Inzlicht and Schmeichel, 2012). This is consistent with research showing that the accessibility of portable devices increases the risk of digital interference, especially in self-regulating learning environments (Gamage and Perera, 2021). Unlike traditional academic environments where structured classroom environments impose external discipline, medical students often learn independently, which makes them more vulnerable to digital disruption (Lei and Zhou, 2012). While portable devices increase access to educational resources, they also increase cognitive switching costs. Several participants said that after long stretches of dense reading “the brain just stops,” prompting a reflexive dive into short-video apps.

Cognitive-load theory predicts that heavy working-memory demand increases susceptibility to salient external stimuli, tilting tired learners toward low-effort rewards instead of another taxing study block (Sweller et al., 2019). Research has shown that mobile-device-based interventions, such as focused apps or screen time management tools, can help balance device dependencies by setting structured digital engagement cycles. By raising awareness of habitual device use and encouraging intentional digital consumption, these strategies can help medical students cope with the challenges of online distraction. Digital traps describe a feedback loop in which initial exposure to digital content triggers extended online conversations, reinforced by algorithmic suggestions and habitual scrolling. Participants reported that initial short social media interruptions tended to escalate into longer browsing sessions. One student described how a single entertainment video resulted in hours of unintentional scrolling, making it difficult to return to learning (Participant E). This pattern reflects research showing that digital platforms exploit cognitive engagement cycles to make disengagement increasingly challenging (van Rensburg, 2018; Dwijuliani et al., 2021; Chung et al., 2022). Research shows that the design of digital platforms significantly affects users’ ability to self-regulate, rather than attributing digital overuse solely to personal discipline issues. Medical students, given their high cognitive workload, may be particularly vulnerable to these reinforcement circuits. Academic institutions should implement digital literacy training that equips students with self-monitoring strategies that enable them to identify and break cycles of habitual engagement. In addition, promoting structured digital breaks-short online leisure time that students intentionally schedule to avoid inadvertent overuse-can serve as a sustainable way to balance productivity and digital relaxation.

Finally, Impact-stage consequences reveal the longer-term cognitive and motivational costs of persistent cyberslacking. The following discussion contextualizes these findings from the existing literature and explores their implications for medical education. The results show that as the relaxation behaviors persist, they gradually weaken students’ learning motivation and perception of learning rewards. Viewed through Self-Determination Theory and temporal-discounting models, this ‘goal-erosion’ pattern emerges when autonomy/competence needs are frustrated and distant, uncertain academic rewards are steeply discounted relative to immediate, low-effort digital gratification (Ryan and Deci, 2020; Steel and König, 2006; Frederick et al., 2002). Previous research has confirmed that intrinsic motivation significantly drives student engagement (Zhao et al., 2011), but this study highlights how frequent digital distractions weaken student motivation over time. This is consistent with the principle of instant gratification theory (Steel and König, 2006), because students can reap immediate rewards from digital content, reinforcing short-term engagement at the expense of long-term academic performance. Long-term exposure to digital entertainment, coupled with delayed gratification of academic achievement, reinforces the tendency to prioritize immediate rewards over long-term learning (Ti et al., 2022). Moreover, uncertainty about academic returns further exacerbates cyberslacking. Unlike social media interactions with instant validation, academic progress in medical education takes years to develop and therefore does not yield immediate returns (Dowling and Brown, 2010). As students experience long-term academic workloads with little short-term reinforcement, they increasingly turn to digital engagement as another form of satisfaction. As students experience long-term academic workloads with little short-term reinforcement, they increasingly turn to digital engagement as another form of satisfaction. This highlights *technology-mediated behavioral addictions* (Griffiths et al., 2017), as repeated exposure to personalized digital content fosters habitual engagement and makes it harder for students to disengage from nonacademic activities. This is consistent with research showing that when the perceived cost–benefit ratio of academic effort becomes unfavorable, students are more likely to disengage and seek other cognitive rewards (Dweck, 1986). Addressing this requires institutional strategies that provide structured and progressive academic reinforcement to sustain long-term engagement. In addition to decreased motivation, academic cyberslacking is strongly associated with cognitive overload. Research shows that students experiencing cognitive fatigue are more susceptible to digital distractions as they seek relief from information saturation (Park et al., 2014). The study supports these findings by showing how prolonged exposure to complex medical content leads to information fatigue, decision-making paralysis, and cognitive fragmentation. Cognitive-load theory predicts that when intrinsic and extraneous loads saturate working-memory capacity, learners default to minimal-effort activities that rapidly relieve mental strain, such as scrolling brief videos (Sweller et al., 2019). Difficulties in processing large amounts of medical knowledge often lead to avoidance behavior, with students turning to digital entertainment rather than making structured learning choices. Previous studies have shown that cognitive overload impairs decision-making, leading to procrastination and task avoidance (Pignatiello et al., 2020). In addition, frequent digital interruptions exacerbate cognitive fragmentation, as students struggle to maintain deep focus amid constant notifications and social media interactions (Dweck, 1986). Limited-capacity attention models and ego-depletion research indicate that repetitive task-switching taxes executive control, progressively lowering the threshold for future distractions and reinforcing a cycle of fragmented focus (Baumeister and Vohs, 2016). This interference impairs their ability to form coherent thought patterns and ultimately reduces learning efficiency. Given these challenges, medical curricula should integrate structured cognitive load management techniques, such as adaptive learning schedules and focused learning environments, to mitigate the negative impact of cyberslacking on information retention. Despite the challenges, cyberslacking is not entirely harmful. The study found a shift in students who strategically adjusted digital engagement to optimize their learning. Research has shown that controlled digital platform engagement can act as a cognitive reset mechanism, allowing students to manage fatigue while maintaining learning efficiency (Liu and Wu, 2023). Selective cyberslacking, for example, allows students to strike a balance between digital breaks and focused learning. This is consistent with research findings that brief, non-academic engagement can enhance cognitive flexibility and improve sustained attention (Baumeister and Vohs, 2016). Moreover, compensatory learning strategies-such as using online educational resources to reinforce missing content-suggest that students are not just passive consumers of digital distractions, but actively integrate them into their learning process. Previous research has highlighted that students who use digital tools for learning adaptation exhibit higher levels of self-regulated learning and resilience (Rezaei et al., 2022). Border policing reinforces this adaptability further, as students establish structured limits on digital engagement. Instead of avoiding online activities altogether, they enforce time limits and self-imposed digital rules, such as learning technical break cycles, to maintain productivity while satisfying their demand for digital engagement (Kwan et al., 2019). These findings suggest that cultivating self-regulating strategies does not eliminate online slackness entirely, but allows students to leverage digital engagement in ways that support, rather than undermine, their academic performance.

## 5 Implications for medical education in the digital age

Drawing on the three-stage grounded theory model identified in this study (Trigger, Sustain, Impact), we propose a stage-sensitive institutional response to academic cyberslacking that integrates motivational support, cognitively efficient instructional design, and technology self-regulation. At the trigger stage, where autonomy deficits, limited instructional interaction, and attention competition from unmanaged notifications invite early off-task digital drift, faculty development should emphasize autonomy-supportive, student-centered, interaction-rich teaching and routine attention-management practices (e.g., structured device pauses, focus/quiet modes) to reduce initial cyberslacking (Dowling and Brown, 2010). To disrupt sustain-stage processes, including algorithmic pull, device dependence, self-control depletion, and cognitive load saturation, programs should embed pre-clinical digital literacy and self-regulation training that teaches notification control, structured study–break cycles, mindful attention skills, and cognitively efficient study strategies that channel device use toward academic purposes as students transition into intensive clinical training (Zhang et al., 2022). To address impact-stage consequences such as motivation decline, reward uncertainty, and cognitive fragmentation, institutions should cultivate learning environments that pair academic-oriented technologies with structured digital recovery intervals, engagement-tracking dashboards, and progress-visibility plus mentorship feedback loops so that students can monitor, interpret, and recalibrate their own digital behavior while sustaining academic focus and well-being across the medical curriculum (Lu et al., 2016).

## 6 Limitations and avenues for future research

Interpretation of these findings should be situated within the specific cultural and institutional context of two medical universities in northwest China, where exam-driven curricula, hierarchical faculty–student relations, and uneven digital integration may shape both the prevalence and the perceived acceptability of academic cyberslacking (Masters et al., 2024). Patterns may differ in systems that vary in instructional language, regulatory structures, resource levels, or online/hybrid delivery models; cross-cultural work on digital distraction and qualitative transferability cautions against direct generalization across settings (Leung, 2015). Our sample comprised only medical students—a high-intensity, professionalizing learner group—so extension to other disciplines or training models should be made cautiously; emerging work shows that medical trainees’ future-oriented expectations are context sensitive (Gotler et al., 2024). We did not systematically model individual differences (e.g., self-regulation skill, smartphone use habits), yet evidence links these attributes to variability in off-task digital behavior and related academic outcomes (Amez and Baert, 2020). Academic stress environments also vary across institutions and may interact with cyberslacking tendencies, suggesting that workload structure could moderate the processes identified in our model (Nweke et al., 2024). Future studies should use multi-site and longitudinal designs that integrate objective usage analytics, motivational and stress measures, and comparative cohorts across disciplines to test the transferability and intervention responsiveness of the trigger–sustain–impact framework proposed here.

## 7 Conclusion

This study explored the mechanism of medical students’ academic cyberslacking and analyzed its trigger factors, duration and long-term consequences. Through a theory-based approach, we identified four main themes. Personal drivers, social and environmental impacts, technological factors, and goal erosion. These factors interact dynamically, leading to the formation and persistence of cyberslacking behavior. Medical students experience a progressive behavioral pattern of cyberslacking, derived from individual and environmental triggers, which are further reinforced by technical engagement and ultimately contribute to long-term goal erosion. Specifically, students are initially distracted by the internet for the following reasons. Autonomy deficits and psychological or physical compensation. Over time, social reinforcement and shifts in academic norms normalize and habituate these behaviors. Technological factors, especially algorithmic reinforcement and device dependency, persist and exacerbate engagement with digital distractions, leading to cognitive overload, motivation decline and fragmentation of learning experiences. Despite the challenges posed by cyberslacking, some students actively develop adaptive strategies such as selective cyberslacking, compensatory learning strategies, and boundary policing to balance digital engagement with academic responsibility. These findings suggest that cyberslacking is not just a time management problem, but rather a complex behavioral adaptation to modern digital learning environments. Tackling this phenomenon requires a multidimensional approach integrating institutional interventions, digital literacy training and self-regulatory strategies to promote healthier digital engagement.

## Data Availability

The original contributions presented in the study are included in the article/supplementary material, further inquiries can be directed to the corresponding author/s.
